# Effects of vitamin D, omega-3 and a simple strength exercise programme in cardiovascular disease prevention: The DO-HEALTH randomized controlled trial

**DOI:** 10.1016/j.jnha.2024.100037

**Published:** 2024-01-09

**Authors:** Stephanie Gaengler, Angélique Sadlon, Caroline De Godoi Rezende Costa Molino, Walter C. Willett, JoAnn E. Manson, Bruno Vellas, Elisabeth Steinhagen-Thiessen, Arnold Von Eckardstein, Frank Ruschitzka, René Rizzoli, José A.P. da Silva, Reto W. Kressig, John Kanis, E. John Orav, Andreas Egli, Heike A. Bischoff-Ferrari

**Affiliations:** a Department of Geriatrics and Aging Research, University of Zurich, Zurich, Switzerland; b Centre on Aging and Mobility, University of Zurich, Zurich, Switzerland; c Department of Nutrition, Harvard T.H. Chan School of Public Health, Boston, MA, USA; d Department of Epidemiology, Harvard T.H. Chan School of Public Health, Boston, MA, USA; e Channing Laboratory, Department of Medicine, Brigham and Women’s Hospital, Boston, MA, USA; f Harvard Medical School, Boston, MA, USA; g Division of Preventive Medicine, Department of Medicine, Brigham and Women’s Hospital, Boston, MA, USA; h Gérontopôle de Toulouse, Institut du Vieillissement, Centre Hospitalo-Universitaire de Toulouse, Toulouse, France; i UMR INSERM 1027, University of Toulouse III, Toulouse, France; j IHU HealthAge, University Hospital Toulouse, France; k Interdisciplinary Lipid Metabolic Center, Charité Universitätsmedizin Berlin, 13353 Berlin, Germany; l Institute of Clinical Chemistry, University of Zurich and University Hospital Zurich, Zurich, Switzerland; m Department of Cardiology, University Heart Center, University Hospital Zurich, Switzerland; n Division of Bone Diseases, Geneva University Hospitals and Faculty of Medicine, Geneva, Switzerland; o Centro Hospitalare Universitário de Coimbra, Coimbra, Portugal; p Coimbra Institute for Clinical and Biomedical Research (ICBR), Faculty of Medicine, University of Coimbra, Coimbra, Portugal; q University Department of Geriatric Medicine FELIX PLATTER and University of Basel, Basel, Switzerland; r Centre for Metabolic Diseases, University of Sheffield Medical School, Sheffield, United Kingdom; s Mary MacKillop Institute for Health Research, Australian Catholic University, Melbourne, Victoria, Australia; t Department of Biostatistics, Harvard T.H. Chan School of Public Health, Boston, MA, USA

**Keywords:** Prevention, Non-pharmaceutical interventions, Lipids, Biomarkers, Hypertension

## Abstract

**Background:**

The effects of non-pharmaceutical interventions in the prevention of cardiovascular diseases (CVD) in older adults remains unclear. Therefore, the aim was to investigate the effect of 2000 IU/day of vitamin D_3_, omega-3 fatty acids (1 g/day), and a simple home strength exercise program (SHEP) (3×/week) on lipid and CVD biomarkers plasma changes over 3 years, incident hypertension and major cardiovascular events (MACE).

**Methods:**

The risk of MACE (coronary heart event or intervention, heart failure, stroke) was an exploratory endpoint of DO-HEALTH, incident hypertension and change in biomarkers were secondary endpoints. DO-HEALTH is a completed multicentre, randomised, placebo-controlled, 2 × 2 × 2 factorial design trial enrolling 2157 Europeans aged ≥70 years.

**Results:**

Participants’ median age was 74 [72, 77] years, 61.7% were women, 82.5% were at least moderately physically active, and 40.7% had 25(OH)D < 20 ng/mL at baseline.

Compared to their controls, omega-3 increased HDL-cholesterol (difference in change over 3 years: 0.08 mmol/L, 95% CI 0.05–0.10), decreased triglycerides (−0.08 mmol/L, (95%CI −0.12 to −0.03), but increased total– (0.15 mmol/L, 95%CI 0.09; 0.2), LDL– (0.11 mmol/L, 0.06; 0.16), and non-HDL-cholesterol (0.07 mmol/L, 95%CI 0.02; 0.12). However, neither omega-3 (adjustedHR 1.00, 95%CI 0.64–1.56), nor vitamin D_3_ (aHR 1.37, 95%CI 0.88–2.14), nor SHEP (aHR 1.18, 95%CI 0.76–1.84) reduced risk of MACE or incident hypertension compared to control.

**Conclusion:**

Among generally healthy, active, and largely vitamin D replete, older adults, treatment with omega-3, vitamin D_3_, and/or SHEP had no benefit on MACE prevention. Only omega-3 supplementation changed lipid biomarkers, but with mixed effects.

**Trial registration ClinicalTrials.gov identifier:**

NCT01745263.

## 1 Introduction

Age is the strongest risk factor for cardiovascular diseases (CVD) [1]. Additionally, older adults are more likely to suffer from functional decline and higher mortality after sustaining a major cardiovascular event [2]. Therefore, efforts to prevent CVD have become a focus of healthy aging campaigns to allow more adults to remain functionally active for longer periods [3].

In recent years, cardiovascular risk stratification models including both blood-based biomarkers (e.g. LDL or HDL levels) and clinical biomarkers (e.g. systolic blood pressure) have been described for older adults, such as SCORE2-OP in Europe [4]. Findings from large landmark studies such as the HYVET and SPRINT trials for hypertension as well as the PROSPER and EWTOPIA trials for lipid lowering agents guide clinicians in their choice of treatment according to the patient’s risk for CVD [[5], [6], [7], [8]]. Still, concerns about the safety and efficacy of these interventions in older adults persist as PROSPER was the only trial specifically designed to test the efficacy of statins older adults, particularly in those older than 75 years [9]. While statins reduced LDL cholesterol levels and major cardiovascular events (MACE) irrespective of age, there is less evidence for a benefit in patients older than 75 years without a history of coronary heart disease or stroke [10]. Further, it has been suggested that statins may increase the risk of diabetes and haemorrhagic stroke, which caused debate on the net cardiovascular benefit of statins for older patients [11]. Also, some of these drugs are at increased risk for inducing side effects in older adults due to age-related variations in pharmacodynamics and pharmacokinetics and drug–drug interactions resulting from polypharmacy [12].

Therefore, in recent years, non-pharmaceutical interventions including marine omega-3 fatty acids and vitamin D_3_ supplementation, as well as physical activity, have attracted increased attention either because of their effect on lipid levels and individual biomarkers of CVD or in the primary prevention of MACE. Indeed, several large cohort studies support physical activity as a major lever in the reduction of MACE [13], while both marine omega-3 and vitamin D_3_ have been suggested to improve lipid profiles and other key pathways influencing MACE risk [[14], [15], [16]]. Furthermore, past studies point towards a role of vitamin D, omega 3 and exercise on blood pressure in older adults [[17], [18], [19]]. In contrast to the lipid-lowering drugs or pharmacological treatments for the prevention of MACE, these interventions are considered safe and affordable for the growing older segment of the population [[20], [21], [22]]. However, evidence from a large randomised controlled trial investigating the effect of non-pharmaceutical interventions on lipid and CVD biomarkers as well as MACE in generally healthy older adults is lacking, mainly because older adults remain under-represented in these studies. As an illustration, a recent systematic review and meta-analysis on the influence of vitamin D supplementation on incident MACE found that only nine out of 21 studies included older adults [23]. Similar observation was reported for studies investigating the role of omega-3 intake on blood pressure [24].

Therefore, the aims of this RCT included investigating the effect of vitamin D_3_, marine omega-3 fatty acids and a strength-training home exercise program (SHEP) on changes in blood lipids and other CVD biomarkers, hypertension and eventually MACE among generally healthy and active community dwelling older adults.

## 2 Methods

### 2.1 Study design and participants

DO-HEALTH was a three-year double-blind, randomised placebo controlled trial conducted in community-dwelling adults aged 70 years and older across seven cities in Europe (Zurich, Basel, Geneva, Innsbruck, Berlin, Toulouse and Coimbra) between December 2012 to November 2014. The study tested three interventions in a 2 × 2 × 2 factorial design: vitamin D_3_ 2000 IU/day vs placebo, omega-3 PUFA of marine origin (1 g 330 mg EPA: 660 mg DHA) vs sunflower oil and 30 min thrice weekly strength exercise training vs 30 min thrice weekly flexibility control training (Table-S2). Sunflower oil was composed of 5% linoleic acid, and less than 2.5 mg/g of oil of EPA and DHA. Detailed composition can be found in the Supplementary material. Study capsules were provided by DSM Nutritional Products Ltd., Switzerland. Participants were randomized to one of the eight treatment combinations (Fig. S1) by stratified block randomization, using study site, fall in the past 12 months, age < or > age 85 years and sex as stratification factors. Both participants and study staff have been blinded, both capsules and exercise kits were provided by a blinded central randomization center. The dose of 2000 IU vitamin D_3_/day was selected, as it has been reported to allow participants to reach blood concentrations of 75 ng/mL, which was to date desirable for fracture prevention [25]. Further, the dose for omega-3s was determined based on studies on secondary prevention of cardiovascular disease [25]. Finally, the strength exercise intervention was validated as effective in the DO-HEALTH pilot trial to prevent hip-fractures [25]. The pre-specified primary aims of the main trial were incidence of non-vertebral fractures, functional decline, changes in systolic and diastolic blood pressure, cognitive decline and rate of infections. Over the three years, participants were followed up by yearly clinical visits and three-monthly telephone calls where infections, medication use, falls, adverse events and health care use were recorded (Fig. S2). Included participants had no major chronic conditions, including no history of myocardial infarction, stroke, transient ischemic attack, angina pectoris, coronary artery intervention or diabetes, at enrolment and in the 5 years prior to the start of the trial. In addition, participants had to be fit enough to come to the study centres and have good cognition (MMSE > 24). Moreover, individuals were excluded who took more than 1000 IU/day of vitamin D_3_ 6 months prior to enrolment or who were unwilling to reduce to 800 IU/day as well as individuals who had taken omega-3 supplements in the 3 months prior to enrolment or were unwilling to stop. Participants taking ≥1000 IU vitamin D_3_/day had to undergo a wash-out period of at least 3 month where they take not more than 800 IU vitamin D_3_/day, details can be found in elsewhere [25]. The adherence to treatment was high with 85.8% of the individuals taking at least 80% of the study pills and 62% performed their exercise program 3 times per week [26]. Further details on the design of the study including masking and randomization, recruitment as well as the study flow can be found elsewhere [25,26]. The study was conducted in accordance with the principles of the declaration of Helsinki and approved by the respective ethical committees and regulatory agencies in the five countries.

### 2.2 Outcomes

#### 2.2.1 Blood biomarkers

The change in the investigated biomarkers were secondary endpoints of DO-HEALTH. For this analysis the whole intention-to-treat DO-HEALTH (n = 2157) study population was used. Blood samples were drawn at yearly visits, immediately processed and aliquoted to 0.5 mL tubes, stored in −80 °C and shipped on a three-monthly basis in dry-ice containers to the central DO-HEALTH biobank at Fisher Clinical Services GmbH (Allschwil, Switzerland). The samples were stored at −80 °C until analysis. Blood biomarkers were all analysed at the Institute for clinical chemistry at the University Hospital Zurich, Switzerland (ISO Certified ISO/IEC 17025) under standardised procedures and with regular quality control. Biomarker concentrations were determined in Li-heparinate plasma. Plasma blood lipids, including total cholesterol, HDL-cholesterol (HDL) and triglycerides (TG) were determined using colorimetric enzymatic assays on a Roche Cobas 8000 with a c701 module. LDL-cholesterol concentrations were calculated from total- and HDL-cholesterol. High-sensitive CRP (hsCRP) concentrations were measured using a particle enhanced immunological turbidity assays on a Roche Cobas 8000 with a c701 module. NT-proBNP and high-sensitive troponin cardiac troponin T (Troponin T) were both assessed with electro chemiluminescence immunoassays (ECLIA) on a Roche Cobas 8000 with a e602 module. Fatty acid measurements were undertaken using sensitive and selective assays based on liquid chromatography combined with mass spectrometry. A table (Table-S4) with limits of detection, calibrated ranges and assays coefficients of variation can be found in the Supplementary information.

#### 2.2.2 Major cardiovascular events

Incident major CVD events (MACE) were an exploratory endpoint of DO-HEALTH and used as a composite (any of the events) individual endpoint and included myocardial infarction, stroke, procedures leading to coronary revascularization, incident congestive heart disease and cardiovascular mortality, which included the following ICD-10 codes: I11, I21:22, I25, I42, I50, I61, I63, I64, Z95 [27]. Medical records were prospectively collected throughout the trial and confirmed by an independent physician endpoint committee. Participants who reported MACE at baseline (protocol deviation) and participants whose MACE status could not be confirmed were excluded from the analysis. The time to event was calculated from the date of randomization until the date of disease onset, death, drop out or end of study.

Incident hypertension was a pre-specified secondary endpoint of DO-HEALTH. Blood pressure was measured at every clinical visit at baseline and year 1–3, after 5-min rest and while seated. The mean of measurement two and three was reported. Incident hypertension was identified as any of these criteria: blood pressure systolic (sBP) ≥140 mmHg and/or diastolic (dBP) ≥90 mmHg, start of an antihypertensive drug, hypertension reported as an adverse event. Participants with prevalent hypertension (using the same definition) were omitted from the analysis.

#### 2.2.3 Other measurements used in the analysis

Physical activity was assessed yearly with an excerpt of the validated Nurses’ Health study (NHS) questionnaire, where participants recorded their usual physical activity during the past year in terms of intensity and duration [28]. This was subsequently converted to metabolic equivalents (METs-h per week). Medication intake was assessed at baseline, every 3 months by phone calls, and every year in the clinical visits. Medications were coded using Anatomical Therapeutic Chemical classification system.

### 2.3 Statistical analysis

Variables were visually inspected for their distribution, non-normal variables are presented as median and interquartile range, normally distributed variables by their mean and standard deviation and categorical variables as percentages. Population characteristics were described by treatment groups. Significant differences between treated and non-treated participants at baseline were tested with Wilcoxon tests, t-tests or chi-square tests, for non-normal, normal and categorical variables, respectively. Non-normal variables are presented as geometric means and geometric standard deviations in the regression tables.

All models were tested for significant treatment interactions. If significant (p < 0.05), an eight-level variable with the different treatment combinations was evaluated. When absence of significant interaction among treatment groups was confirmed, the main effects for the three treatments were examined in the models.

The treatment effect on incident MACE and incident hypertension was assessed using the Cox-proportional hazards model with study site specified as strata and covariate adjustment for sex, previous fall, BMI, age and spline at age 85. The proportional hazards assumption was tested using the proportionality test function in the PROC *phreg*. To do so, the interaction of the logarithm of follow-up time and the variables in the model were added to the model of interest and their significance and the overall significance of the test was evaluated. With regard to power, with 81 MACE events, the trial would need a hazard ratio <0.53 in order to be detected with 80% power. Proportionality of hazards assumption was tested by the inclusion of time dependent covariates in the regression model. Significant time dependent predictors would violate the model assumptions.

The change of biomarkers over time was assessed using linear-mixed models adjusted for baseline biomarker concentration, sex, age and a linear spline for age with a knot at age 85 to control for the different aging rate in older age, baseline BMI and change in BMI over time as a time-varying covariate, prior fall and study site. Residuals of the models were assessed for their normality (mean of 0 and standard deviation 1). Further the variance between treatment groups was assessed. Analyses were undertaken with R (v 4.0.2) in RStudio (v 1.2.1578) and SAS (v 9.4).

## 3 Results

### 3.1 Baseline characteristics

The sample size for the biomarker analyses was 2157 participants, for the incident MACE analysis, the sample size was 2089 after excluding unconfirmed reported events and baseline events. In both cases, the median follow-up time was 2.99 years. Baseline characteristics were balanced across the groups following randomization, unless indicated in the footnote of Table 1. Participants’ median age was 74 [72, 77] years and women represented 61.7% (n = 1331) of the sample. Mean systolic and diastolic BP levels were 143.53 (18.35) mmHg and 75.88 (10.04) mmHg, respectively. Lipid profile showed triglycerides levels of 1.04 [0.82, 1.35] mmol/L, total cholesterol of 5.58 (1.07) mmol/L, HDL of 1.71 (0.46) mmol/L and LDL of 3.35 (0.95) mmol/L. Among the behavioural CVD risk factors, more than half of the participants (52.3%, n = 1128) reported being active at least 3 times a week and only a small minority (5.8%, n = 126) were current smokers. About one in four (26.3%, n = 568) participant was taking a lipid-lowering medication and nearly half of the participants (49.6%, n = 1069) took at least one antihypertensive drug. Finally, mean 25(OH)D levels in blood were 22.39 (8.42) ng/mL while mean DHA and EPA levels were 78.08 (36.91 mg/L and 30.85 (20.71) mg/L, respectively (Table 1, or Supplementary Table-S1 for the MACE sample).

**Table 1:** Population characteristics of the DO-HEALTH population used for the biomarker analyses.

	Overall	Vitamin D_3_	No vitamin D_3_	Omega-3	No omega-3	SHEP	No SHEP
	N = 2157	N = 1076	N = 1081	N = 1073	N = 1084	N = 1081	N = 1076
***Demographics***							
Age [years]	74.00 [72.00, 77.00]	74.00 [71.75, 77.00]	74.00 [72.00, 77.00]	74.00 [71.00, 77.00]	74.00 [72.00, 77.00]	74.00 [72.00, 77.00]	74.00 [71.00, 77.00]
Women n (%)	1331 (61.7)	667 (62.0)	664 (61.4)	668 (62.3)	663 (61.2)	665 (61.5)	666 (61.9)
Education [years]	12.64 (4.31)	12.67 (4.45)	12.61 (4.16)	12.60 (4.20)	12.68 (4.41)	12.63 (4.24)	12.65 (4.37)
***Clinical variables***							
Systolic BP [mmHg]	143.53 (18.35)	144.15 (18.56)	142.92 (18.12)	143.20 (18.29)	143.86 (18.41)	143.69 (18.52)	143.37 (18.19)
Diastolic BP [mmHg]	75.88 (10.04)	76.03 (10.10)	75.73 (9.99)	75.82 (9.72)	75.94 (10.36)	75.91 (10.27)	75.85 (9.82)
Self-reported hypertension — n (%)	844 (39.2)	427 (39.7)	417 (38.6)	414 (38.6)	430 (39.7)	405 (37.5)	439 (40.8)
BMI [kg/m^2^]	26.32 (4.29)	26.49 (4.40)	26.16 (4.17)	26.29 (4.24)	26.36 (4.34)	26.27 (4.22)	26.38 (4.36)
Diabetes type 2 — n (%)	150 (7.0)	81 (7.5)	69 (6.4)	73 (6.8)	77 (7.1)	68 (6.3)	82 (7.6)
CVD history — n (%)	88 (4.3)	45 (4.4)	43 (4.2)	41 (4.0)	47 (4.6)	48 (4.6)	40 (3.9)
***Behavioral risks***							
Smoking status n (%)							
Former	671 (31.1)	339 (31.5)	332 (30.7)	337 (31.4)	334 (30.8)	351 (32.5)	320 (29.7)
Never	1360 (63.1)	674 (62.6)	686 (63.5)	672 (62.6)	688 (63.5)	672 (62.2)	688 (63.9)
Smoker	126 (5.8)	63 (5.9)	63 (5.8)	64 (6.0)	62 (5.7)	58 (5.4)	68 (6.3)
Physical activity							
None n (%)	375 (17.4)	207 (19.2)	168 (15.5)	190 (17.7)	185 (17.1)	179 (16.6)	196 (18.2)
1–2 time per week	652 (30.2)	318 (29.6)	334 (30.9)	311 (29.0)	341 (31.5)	323 (29.9)	329 (30.6)
≥3 times per week	1128 (52.3)	550 (51.1)	578 (53.5)	570 (53.1)	558 (51.5)	578 (53.5)	550 (51.1)
NHS-METs [METs h/week]	36.95 (33.09)	35.39 (32.01)	38.50 (34.07)^c^	38.17 (34.12)	35.73 (32.01)	37.01 (33.44)	36.88 (32.75)
Fat intake [g/day]^a^, ^b^	95.53 [71.64, 124.46]	96.62 [72.32, 123.21]	94.55 [70.98, 126.02]	95.55 [72.12, 125.14]	95.42 [71.28, 123.21]	96.19 [72.04, 124.71]	94.97 [70.97, 123.86]
***Drug intake***							
Lipid lowering drugs							
Any — n (%)	568 (26.3)	304 (28.3)	264 (24.4)	267 (24.9)	301 (27.8)	291 (26.9)	277 (25.7)
Statin — n (%)	541 (25.1)	287 (26.7)	254 (23.5)^d^	251 (23.4)	290 (26.8)	275 (25.4)	266 (24.7)
Fibrates — n (%)	26 (1.2)	13 (1.2)	13 (1.2)	16 (1.5)	10 (0.9)	14 (1.3)	12 (1.1)
Bile acid	1 (0.0)	0 (0.0)	1 (0.1)	0 (0.0)	1 (0.1)	0 (0.0)	1 (0.1)
Sequestrant — n (%)							
Other n (%)	29 (1.3)	18 (1.7)	11 (1.0)	17 (1.6)	12 (1.1)	15 (1.4)	14 (1.3)
Antihypertensive drugs	1069 (49.6)	548 (50.9)	521 (48.2)	509 (47.4)	560 (51.7)	516 (47.7)	553 (51.4)
Sex hormone use n (%)	138 (6.4)	65 (6.0)	73 (6.8)	69 (6.4)	69 (6.4)	62 (5.7)	76 (7.1)
***Laboratory values at baseline***							
25-hydroxyvitamin D -(25(OH)D) [ng/mL]	22.39 (8.42)	22.36 (8.40)	22.42 (8.45)	22.39 (8.42)	22.39 (8.42)	22.79 (8.58)	21.99 (8.25)
	872 (40.7)	427 (40.1)	445 (41.4)	422 (39.7)	450 (41.8)	422 (39.4)	450 (42.1)
Triglycerides [mmol/]^a^	1.04 [0.82, 1.35]	1.05 [0.83, 1.37]	1.02 [0.82, 1.32]	1.03 [0.81, 1.35]	1.04 [0.83, 1.34]	1.02 [0.81, 1.31]	1.05 [0.83, 1.38]
Total cholesterol [mmol/L]	5.58 (1.07)	5.54 (1.07)	5.62 (1.07)	5.60 (1.05)	5.56 (1.09)	5.59 (1.06)	5.57 (1.08)
HDL-cholesterol [mmol/L]	1.71 (0.46)	1.70 (0.47)	1.72 (0.46)	1.71 (0.46)	1.71 (0.47)	1.73 (0.47)	1.69 (0.46)^f^
LDL-cholesterol [mmol/L]	3.35 (0.95)	3.31 (0.96)	3.38 (0.95)	3.37 (0.94)	3.32 (0.97)	3.35 (0.96)	3.34 (0.95)
Non-HDL cholesterol [mmol/L]	3.87 (1.03)	3.85 (1.02)	3.90 (1.03)	3.90 (1.01)	3.85 (1.04)	3.87 (1.03)	3.88 (1.03)
Homocysteine [μmol/L]^a^	13.8 [11.50, 16.30]	13.8 [11.50, 16.20]	13.75 [11.40, 16.42]	13.8 [11.50, 16.40]	13.7 [11.40, 16.20]	13.8 [11.60, 16.00]	13.6 [11.30, 16.60]
NT-proBNP [ng/L]^a^	112 [66.00, 186.00]	112 [66.00, 189.00]	112 [67.00, 184.00]	110 [67.00, 189.00]	115 [66.00, 184.00]	114 [66.00, 191.00]	111 [66.00, 182.00]
Troponin T [ng/L]^a^	6 [4.00, 10.00]	7 [3.98, 10.00]	6 [4.00, 10.00]	6 [3.93, 10.00]	7 [4.07, 10.00]^e^	6 [3.95, 10.00]	6 [4.03, 10.00]
hs-CRP[mg/L]^a^	1.5 [0.80, 2.90]	1.6 [0.80, 2.80]	1.5 [0.80, 3.00]	1.6 [0.80, 3.00]	1.5 [0.80, 2.90]	1.5 [0.80, 2.80]	1.6 [0.80, 3.00]
DHA [mg/L]	78.08 (36.91)	78.11 (37.88)	78.05 (35.93)	78.88 (37.20)	77.30 (36.62)	78.15 (36.48)	78.01 (37.35)
EPA [mg/L]	30.85 (20.71)	30.61 (21.54)	31.10 (19.85)	30.78 (19.98)	30.93 (21.40)	30.64 (20.79)	31.06 (20.63)

Abbreviation: SHEP- simple home based strength exercise program; BP- blood pressure; NHS-METs- Nursses' Health Study - Metabolic equivalents; hs-CRP- high sensitvity C-reactive protein; DHA- docosahexaenoic acid; EPA- eicosapentaenoic acid.

a Non-normal median and interquartile range are presented and -test used to test difference between groups.

b Participants with values above 4000 kcal/day for women and 5000 kcal/day for men omitted as over reporting (n = 51).

c Difference between two groups: p-value = 0.029.

d Difference between two groups p-value = 0.049.

e Difference between two groups p-value = 0.08.

f Difference between two groups p-value = 0.047.

### 3.2 Effect on the lipid profile and cardiovascular disease biomarkers

**Fig. 1:**
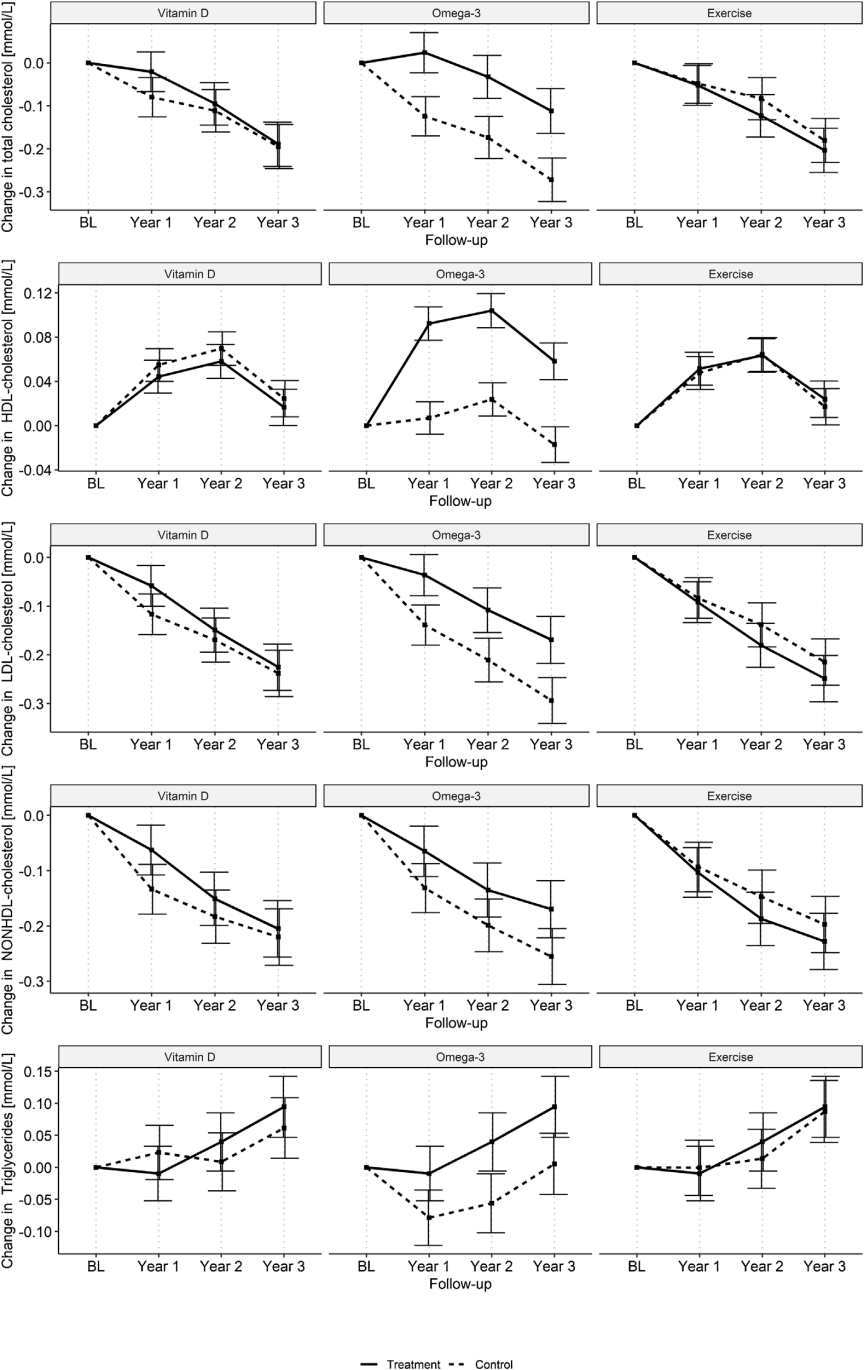
Yearly adjusted least square mean change from baseline and their 95% confidence interval of the CVD risk factors retrieved from the linear mixed effect models. Triglycerides concentrations in the treatment groups were compared to placebo, all others were compared to not receiving the treatment.

**Fig. 2:**
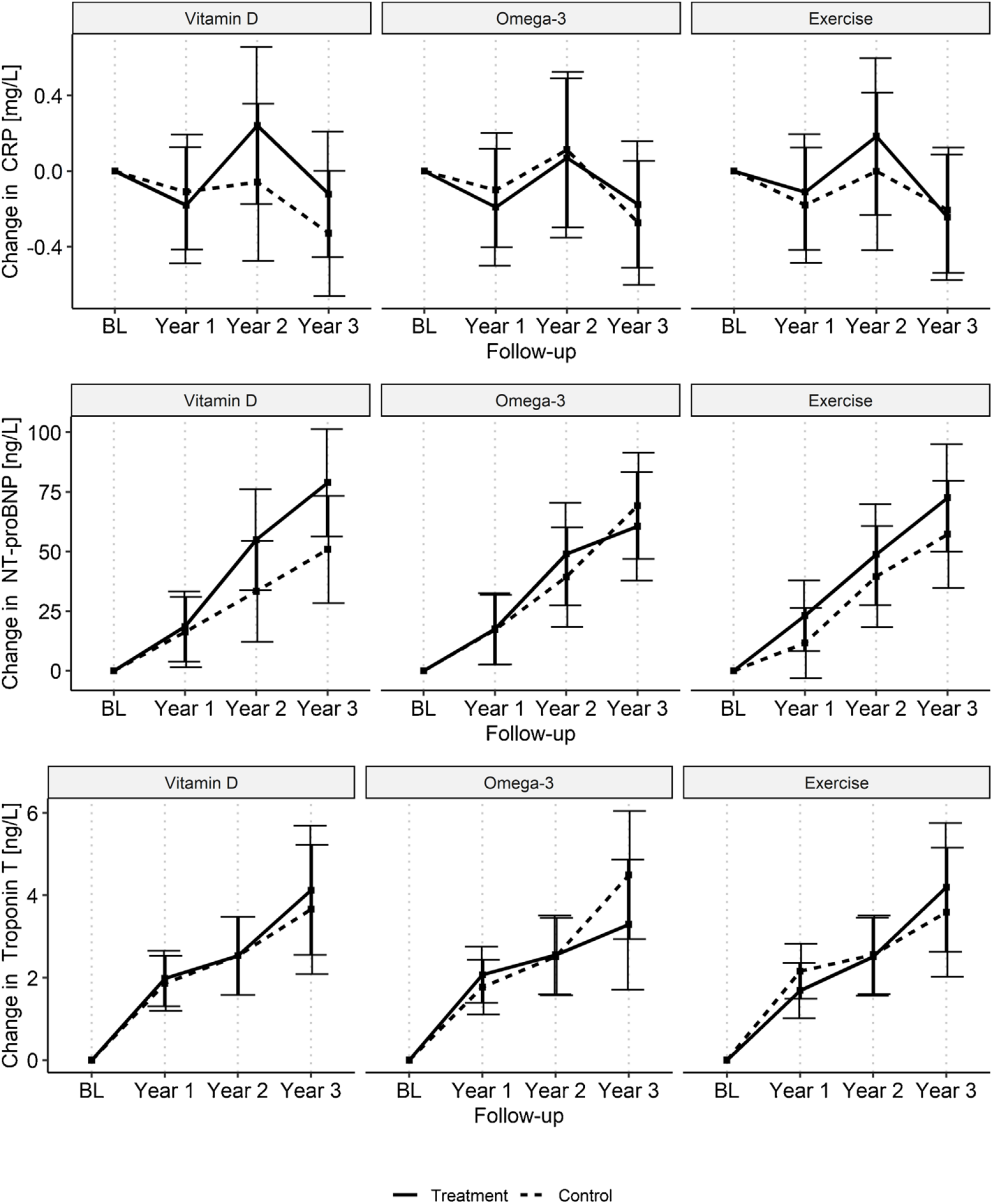
Yearly adjusted least square mean change from baseline and their 95% confidence interval of the CVD risk markers retrieved from the linear mixed effect models.

For the lipid profile, vitamin D_3_ and SHEP interventions did not yield any significant changes from baseline over time (Table 2). However, we found that omega-3 supplementation increased HDL levels (difference in change from baseline over 3 years: 0.08 mmol/L, 95% CI 0.06–0.10 mmol/L) and decreased triglycerides levels (difference in change from baseline over 3 years: −0.08 mmol/L, 95% CI −0.12 to −0.03 mmol/L) over time compared to no omega-3 and placebo, respectively. Compared to the sunflower oil control group, the omega-3 intervention group showed an increase in total cholesterol, LDL-cholesterol, and non-HDL cholesterol over time (Fig. 1). We did not find any statistically significant changes from baseline over time for any of NT-proBNP, troponin T, or hs-CRP for the three intervention groups (Table 2 and Fig. 2).

**Table 2:** Least Square Means (LSM) and their differences for each treatment group of the different cardiovascular health biomarkers from linear mixed effect regression models.

Biomarkers	Event	Vitamin D_3_ (n = 1069)	No vitamin D_3_ (n = 1072)	Difference (95%CI), p-value	Omega-3 (n = 1064)	No omega-3 (n = 1077)	Difference (95%CI), p-value	SHEP (n = 1072)	No SHEP (n = 1069)	Difference (95%CI), p-value
**CVD risk factors**										
Total cholesterol [mmol/L]	Baseline mean (SD)	5.54 (1.07)	5.62 (1.07)		5.60 (1.05)	5.56 (1.09)		5.59 (1.06)	5.57 (1.08)	
	Change across all 3 years	−0.09 (−0.13; −0.06)	−0.13 (−0.17; −0.1)	0.04 (−0.01; 0.09), p = 0.115	−0.03 (−0.07; 0)	−0.19 (−0.22; −0.15)	0.15 (0.11; 0.2), p < 0.001	−0.11 (−0.15; −0.08)	−0.11 (−0.14; −0.07)	−0.01 (−0.05; 0.04), p = 0.807
HDL-cholesterol [mmol/L]	Baseline mean (SD)	1.70 (0.47)	1.72 (0.46)		1.71 (0.46)	1.71 (0.47)		1.73 (0.47)	1.69 (0.46)	
	Change across all 3 years	0.04 (0.03; 0.05)	0.05 (0.04; 0.06)	−0.01 (−0.03; 0.01), p = 0.3	0.09 (0.07; 0.1)	0 (−0.01; 0.02)	0.08 (0.06; 0.1), p < 0.001	0.05 (0.03; 0.06)	0.04 (0.03; 0.06)	0 (−0.01; 0.02), p = 0.633
LDL-cholesterol [mmol/L]	Baseline mean (SD)	3.31 (0.96)	3.38 (0.95)		3.37 (0.94)	3.32 (0.97)		3.35 (0.96)	3.34 (0.95)	
	Change across all 3 years	−0.14 (−0.17; −0.1)	−0.18 (−0.21; −0.15)	0.04 (0; 0.08), p = 0.066	−0.1 (−0.13; −0.07)	−0.21 (−0.24; −0.18)	0.11 (0.07; 0.16), p < 0.001	−0.16 (−0.19; −0.13)	−0.15 (−0.18; −0.12)	−0.01 (−0.06; 0.03), p = 0.534
Non-HDL cholesterol [mmol/L]	Baseline mean (SD)	3.85 (1.02)	3.90 (1.03)		3.90 (1.01)	3.85 (1.04)		3.87 (1.03)	3.88 (1.03)	
	Change across all 3 years	−0.13 (−0.17; -0.1)	−0.18 (−0.21; −0.15)	0.05 (0; 0.1), p = 0.036	−0.12 (−0.15; −0.08)	−0.19 (−0.23; −0.16)	0.07 (0.03; 0.12), p = 0.002	−0.16 (−0.2; −0.13)	−0.15 (−0.18; −0.12)	−0.01 (−0.06; 0.03), p = 0.615

		Vitamin D (n = 272)	Placebo (n = 269)	Difference (95%CI)	Omega-3 (n = 266)	Placebo (n = 269)	Difference (95%CI)	SHEP (n = 264)	Placebo (n = 269)	Difference (95%CI)
Triglycerides [mmol/L]^a^	Baseline geometric mean (GSD)	1.07 (1.46)	1.08 (1.5)		1.06 (1.46)	1.08 (1.5)		1.04 (1.43)	1.08 (1.5)	
	Change across all 3 years	0.04 (0; 0.07)	0.03 (0; 0.07)	0 (-0.04; 0.05), p = 0.921	−0.04 (−0.08; −0.01)	0.03 (0; 0.07)	−0.08 (−0.12; −0.03), p = 0.002	0.04 (0; 0.07)	0.03 (0; 0.07)	0 (−0.05; 0.05), p = 0.968
**CVD risk markers**										
hs-CRP [mg/L]	Baseline mean (SD)	1.66 (2.56)	1.65 (2.57)		1.68 (2.61)	1.64 (2.52)		1.64 (2.6)	1.68 (2.54)	
	Change across all 3 years	−0.01 (−0.24; 0.21)	−0.14 (−0.37; 0.09)	0.12 (−0.18; 0.43), p = 0.426	−0.1 (−0.33; 0.13)	−0.06 (−0.28; 0.17)	−0.04 (−0.34; 0.27), p = 0.815	−0.04 (−0.26; 0.19)	−0.12 (−0.35; 0.11)	0.08 (−0.22; 0.39), p = 0.592
NT-proBNP [ng/L]	Baseline geometric mean(GSD)	117.43 (2.47)	117.95 (2.39)		117.6 (2.43)	117.78 (2.43)		119.82 (2.51)	115.59 (2.35)	
	Change across all 3 years	45.72 (30.09; 61.34)	39 (23.44; 54.56)	6.71 (−13.24; 26.67), p = 0.509	41.57 (25.77; 57.36)	43.15 (27.75; 58.55)	−1.58 (−21.56; 18.4), p = 0.877	48.76 (33.11; 64.42)	35.95 (20.41; 51.49)	12.81 (−7.16; 32.78), p = 0.208
Troponin T [ng/L]	Baseline geometric mean (GSD)	6.57 (2.01)	6.37 (1.93)		6.19 (1.94)	6.75 (1.99)		6.49 (1.96)	6.44 (1.98)	
	Change across all 3 years	2.87 (2.24; 3.5)	2.74 (2.12; 3.36)	0.13 (−0.63; 0.89), p = 0.737	2.83 (2.2; 3.46)	2.78 (2.16;3.4)	0.05 (−0.71; 0.81), p = 0.892	2.64 (2.01; 3.27)	2.97 (2.35; 3.6)	−0.33 (−1.09; 0.43), p = 0.39

Note: Models were adjusted for baseline biomarker concentration, visit, baseline body mass indesx (BMI) and change of BMI from baseline, prior fall, sex, study site, age, and a spline at age 85. The change yearly change was calculated using the interaction between treatment and visit. The overall change across three years was assessed without interaction, due to lack of significance of the interaction term.

Abbreviations: CI- confidence interval; SD- standard deviation; GSD- geometric standard deviation; hs-CRP- high sensitivity C-reactive protein.

a For triglycerides there was a significant treatment interaction (p < 0.1). Therefore, we included treatment indicators for each of the 8 combinations of treatments in the regression models and each intervention group (with neither of the other two interventions present) was compared to the 270 participants who received none of the 3 intervention.

### 3.3 Major cardiovascular events

Population characteristics of the MACE population subset can be found in the Supplement (Table-S1). There were 23 deaths in the MACE study population of which 20 were non-MACE related. A total of 81 (3.95%) MACE (based on composite score) occurred during the study period. Based on the main effects analysis, we did not find any statistically significant benefit of any of the three interventions with regard to the risk of MACE: omega-3 versus no omega-3 (adjusted hazard ratio (aHR) = 1.00, 95% CI 0.64–1.56), nor vitamin D_3_ versus no vitamin D_3_ (aHR = 1.37, 95% CI 0.88–2.14), SHEP versus control exercise (aHR = 1.18, 95% CI 0.76–1.84). Also, no significant effect of any of the interventions on MACE as individual endpoints was seen. Excluding participants with prevalent hypertension at baseline, there was also no benefit of any of the three interventions on incident hypertension: omega-3 versus no omega-3: aHR = 0.83, 95% CI 0.60–1.14; vitamin D_3_ versus no vitamin D_3_: aHR = 1.05, 95% CI 0.76–1.44; SHEP versus control exercise: aHR = 1.17, 95% CI 0.85–1.62) (Table 3).

**Table 3:** Adjusted hazard ratios and 95% confidence intervals for the outcomes.

Outcome	Event frequency	Omega-3 vs no omega-3		Vitamin D_3_ vs no vitamin D_3_		SHEP vs control	
		HR (95%CI)	p-Value	HR (95%CI)	p-Value	HR (95%CI)	p-Value
MACE^a^	81/2089	1.00 (0.64; 1.56)	0.992	1.37 (0.88; 2.14)	0.167	1.18 (0.76; 1.84)	0.457
Myocardial infarction^b^	17/2026	0.89 (0.34; 2.32)	0.817	1.41 (0.53; 3.71)	0.489	1.10 (0.42; 2.86)	0.845
Stroke^b^	30/2039	1.32 (0.64; 2.73)	0.447	0.92 (0.45; 1.88)	0.8113	1.00 (0.49; 2.05)	1.0
Hypertension^a^	159/536	0.83 (0.60; 1.14);	0.255	1.05 (0.76; 1.44)	0.781	1.17 (0.85; 1.62)	0.328

Note: Disease definitions (ICD 10-Code) major cardiovascular events (MACE): any confirmed MACE including following ICD10 codes: I11, I21:22, I25, I42, I50, I61, I63, I64, Z95.

Myocardial infarction: acute myocardial infarction (I21), subsequent myocardial infarction (I22), certain current complications following an acute myocardial infarction (this is not a cardiovascular event itself but the code requires a previous myocardial infarction).

Stroke: intracerebral haemorrhage (I61), cerebral infarction (I63), stroke not specified (I64).

Hypertension: a case is defined if any of the following criteria are met: started medication for HT, has systolic blood pressure (SBP) ≥140 mmHg or diastolic blood pressure (DBP) ≥90 mmHg, reports hypertension in adverse events (I10:I15).

Additionally:

a Main effects cox proportional hazards model, stratified for study site, adjusted for sex, age, prior fall, treatment, body mass index, spline at age 85.

b Main effects cox proportional hazards model stratified for study site included was anyone with a baseline medication for hypertension, elevated SBP or DBP or diagnosis is excluded from the analysis. Participants with prevalent hypertension were excluded for this analysis.

## 4 Discussion

In the 3-year DO-HEALTH trial enrolling 2157 generally healthy and active adults aged 70 years and older, we found that omega-3 supplementation decreased triglycerides levels and increased HDL levels when compared to placebo that contained sunflower oil; however, omega-3 also decreased LDL, non-HDL and total cholesterol to a lesser extent than placebo suggesting mixed benefits of omega-3s on blood lipids. Also, none of the treatments had an effect on troponin or NT-proBNP. Finally, we observed no benefit of any of the three interventions tested (1 g omega-3/day, 2000 IU vitamin D_3_/day, or SHEP) on the prevention of MACE or incident hypertension.

For omega-3 supplementation, our findings are generally consistent with the literature with regard to the prevention of MACE, keeping in mind that our statistical power was low. Since the promising results from the 1999 GISSI-Prevenzione trial, reporting reduced MACE and reduced mortality risks in individuals receiving omega-3 supplementation, the more recent trials have largely failed to replicate and confirm those findings. Summarizing these later trials, a Cochrane systematic review and meta-analysis of more than 79 RCTs concluded that the evidence for omega-3 in the prevention of MACE is weak [15]. This was further supported by the recently published US VITAL trial enrolling 25,871 participants age 50 and older, which found a small but statistically non-significant reduction in the primary prevention of MACE with daily supplementation with 1 g of omega-3 compared to placebo [29]. However, VITAL reported a 28% reduction for their secondary endpoint of myocardial infarction [29]. The GISSI and VITAL trials tested EPA:DHA in a 1.2:1 ratio (1 g/d total), while DO-HEALTH tested 1 g/d with an EPA:DHA ratio of 0.5:1 [30,31]. Only the recent REDUCE-IT trial found a 25% reduction in MACE with daily supplementation of omega-3 compared to placebo [32]. This may in part be explained by the pre-selection on CVD risk, higher dose of omega-3 and different composition of omega-3 fatty acids tested, as well as potentially adverse effects of mineral oil used as the placebo in REDUCE-IT. Both DO-HEALTH and VITAL tested 1 g of omega-3 per day including both DHA and EPA in primary prevention, while REDUCE-IT tested 4 g of omega-3 per day including only EPA in participants with increased CVD risk [32]. Conversely, the latest STRENGTH trial enrolling 13,078 participants with high CVD risk, found that 1 g of omega-3 per day including both DHA and EPA, did not reduce MACE compared to placebo [33]. Thus, overall, DO-HEALTH is in line with trial findings that 1 g of omega-3 per day does not reduce MACE, adding information on the age-group 70 years and older, which represented only a minor fraction of the participants in the earlier trials of omega-3 [30,34,35].

For the vitamin D_3_ intervention, we found no significant benefit on CVD biomarkers or MACE. While this is in contrast with mechanistic studies that support a possible benefit of vitamin D_3_ on vascular resistance [36], and observational studies that found an inverse association between 25-hydroxyvitamin D levels and MACE [37,38], our findings are in line with the large VITAL, VIDA (vitamin D assessment), which did not show any benefit of vitamin D_3_ supplementation on MACE [31,39]. Similarly, the recent D-HEALTH trial failed to show that a monthly 60,000 UI vitamin D_3_ supplementation reduced the risk of major cardiovascular events [40]. Relevant for the planning of future trials, however, it is important to note that DO-HEALTH, VITAL, and most other vitamin D trials have enrolled largely vitamin D replete participants, and both DO-HEALTH and VITAL did not prohibit the intake of 800 IU per day vitamin D_3_ supplementation outside the study medication. Both VITAL and DO-HEALTH also highlight that daily vitamin D_3_ supplementation of 2000 IU per day does not result in adverse cardiovascular events.

Also for the exercise intervention, we found no benefit on any CVD outcome tested. Importantly, however, our results do not question the abundance of data advocating the importance of exercise in preventing CVD [41]. Alternatively, we suggest two possible explanations for the lack of benefit of the DO-HEALTH exercise program on MACE [42]. First, the simple home exercise program tested in DO-HEALTH may not have been intense enough to influence CVD outcomes [43]. Second, 83% of participants enrolled in DO-HEALTH were already moderately to highly physically active at the start of the trial and kept their physical activity level stable regardless of treatment group. This may also explain, why we did not see any benefits of the exercise program on lipid profiles, and other biomarkers of CVD risk compared to the control exercise group. Similarly, the recent Generation 100 trial found no benefit on MACE in individuals aged 70 years and older undergoing a moderate to high intensity exercise program, and the authors noted that participants were already healthy and active at baseline [44].

With regard to biomarkers of CVD, NT-proBNP is a well-established diagnostic, predictive and prognostic marker of heart failure [45]. In DO-HEALTH, none of the interventions changed NT-proBNP longitudinally. For vitamin D, observational studies have suggested an inverse correlation between 25-hydroxyvitamin D and NT-proBNP levels, however, RCT data on the benefits of vitamin D supplementation on NT-proBNP levels is lacking [46,47]. Likewise, past studies, such as the Alpha Omega trial, have failed to show an effect of omega-3 supplementation on NT-proBNP levels [48].

Our study has several strengths. The target population was comprised of generally healthy and active older adults, a population which is often neglected in clinical trials. Further, adherence to treatment was high with 85.8% of the individuals taking at least 80% of the study pills and 62% performed their exercise program 3 times per week [26]. Finally, DO-HEALTH tested two of the same interventions (1 g of omega-3, 2000 IU vitamin D_3_) as the much larger VITAL study and our finding on MACE for both nutrients is in line with the VITAL findings, although in VITAL a significant benefit of omega-3 on myocardial infarction was observed [29].

There are also limitations. DO-HEALTH was not powered to detect significant reduction in MACE. With 81 MACE, the study was underpowered for the composite outcome and for individual MACE, and therefore addressed as an exploratory analysis in DO-HEALTH. Additionally, the three-year follow-up may have been too short to detect an effect on MACE in this generally healthy and active study population. Finally, the DO-HEALTH study population was largely vitamin D replete and over 80% were at least moderately physically active, which may have introduced a conservative bias for the vitamin D_3_ and SHEP interventions tested.

In summary, supplemental omega-3s fatty acids had mixed effects on blood lipids, and vitamin D and SHEP had no effect on any CVD biomarkers in this population. Clinical relevance of the effect of omega-3 on triglycerides may be insignificant. Further, our findings in generally healthy and active community-dwelling older adults did not support a benefit of supplementation with daily 2000 IU vitamin D_3_ or 1 g of omega-3 fatty acids or SHEP in preventing MACE or incident hypertension. This lack of effect may be related to the limited number of endpoints and duration of the intervention.

## Funding

The study was funded by the Seventh Framework Program of the 10.13039/501100000780European Commission(grant agreement 278588; Principal Investigator: Heike A. Bischoff-Ferrari, MD, DrPH), the 10.13039/501100006447University of Zurich(Chair for Geriatric Medicine and Aging Research), DSM Nutritional Products, Roche,NESTEC,Pfizer, andStreuli Pharma.

## Author’s contributions

Conceptualization: SG, AS, CdG, WW, JEM, EST, AvE, FR, JdS, EJO, HABF.

Data curation: SG, AS, CdG, BV, RR, JdS, RK, AE.

Funding: HABF, BV.

Investigation: AS, BV, EST, RR, JdS, RK, HABF.

Project administration: AE, JdS, RK.

Methodology: SG. AS, CdG, WW, JEM, BV, EST, AvE, FR, RK, JK, EJO, HABF.

Formal analysis: SG, AS, CdG, WW, EJO, HABF.

Visualization: SG.

Supervision: WW, JEM, EST, AvE, FR, JK, EJO, HABF.

Writing – original draft: SG, AS, CdG, HABF.

Writing – review and editing: SG, AS, CdG, WW, JEM, BV, EST, AvE, FR, RR, JdS, RK, JK, EJO, AE, HABF.

## Data availability

In a first step, no data will be made available to researchers external to DO-HEALTH Research Group to allow primary researchers to fully exploit the dataset. The data will be shared in a second step according to a controlled access system.

## Conflicts of interest

Stephanie Gaengler has no financial disclosures.

Angelique Sadlon has no financial disclosures.

Caroline De Godoi Rezende Costa Molino has no financial disclosures.

Walter C. Willett has no financial disclosures.

JoAnn E. Manson has no financial disclosures.

Bruno Vellas reported receipt of grants and personal fees from Nestlé and grants from DSL.

Elisabeth Steinhagen-Thiessen has no financial disclosures.

Arnold von Eckardstein reported funding for Technicians by the European Commission, grants from the Swiss National Science foundation, the Swiss Heart Foundation, a role as a secretary of the European Atherosclerosis Society (until 2020) and to be an executive board member of the Swiss Atherosclerosis Society.

Dr. Ruschitzka reported travel grants from AstraZeneca (IMC/A+Science AB), Boehringer Ingelheim, Centro Hospitaler de Vila Nova de Gaia, C.T.I. GmbH, European Society of Cardiology, Novartis, Spektar Putovanja. He also reports Secretarial and administrative support of the HFA President/Past-President 2018–2020 for the European Society of Cardiology.

Rene Rizzoli reported receipt of personal fees from Abiogen and being part of the executive committee of the International Osteoporosis foundation and chairing the scientific board of the European Society for clinical and Economic Aspects of Osteoporosis.

Jose AP da Silva reported receipt of personal fees from Forum D and being publicly involved in the promotion of awareness on the potential importance of vitamin D in the health of individuals and populations and support for lectures from Jaba Recordati.

Reto W. Kressig has no financial disclosures.

John Kanis reports honrary role in the International Osteoporosis Foundation, Patron Osteoporosis 2000, National Osteoporosis Guidelines Group and the honorary presidency of the European Society for Clinical and Economic Aspects of Osteoporosis and Osteoarthritis. Dr Kanis is an architect for FRAX without payment.

Endel J. Orav has no financial disclosures.

Andreas Egli has no financial disclosures.

Heike A. Bischoff-Ferrari reported receipt of nonfinancial support from Roche Diagnostics, grants from Pfizer and Vifor, funds for lectures from Vifor, and OM Pharma, and a payments for expert testimony by BioMed.

## Declaration of interests

The authors declare the following financial interests/personal relationships which may be considered as potential competing interests:

Heike Bischoff-Ferrari reports financial support was provided by European Commission. Heike Bischoff-Ferrari reports financial support was provided by DSM. Heike Bischoff-Ferrari reports financial support was provided by Pfizer Inc. Heike Bischoff-Ferrari reports was provided by Streuli. Heike Bischoff-Ferrari reports financial support was provided by Nestec SA. Heike Bischoff-Ferrari reports equipment, drugs, or supplies was provided by Roche. Heike Bischoff-Ferrari reports a relationship with Vifor Pharma Switzerland SA that includes: speaking and lecture fees. Heike Bischoff-Ferrari reports a relationship with OM_Pharma that includes: speaking and lecture fees. If there are other authors, they declare that they have no known competing financial interests or personal relationships that could have appeared to influence the work reported in this paper.
