# Supplementary material for: Effects of vitamin D, omega-3 and a simple strength exercise programme in cardiovascular disease prevention: The DO-HEALTH randomized controlled trial
Source: J Nutr Health Aging. 2024 Jan 9;28(2):100037. doi: 10.1016/j.jnha.2024.100037 (PMC12877244; doi:10.1016/j.jnha.2024.100037)
Supplement: Supplementary file 1 [file mmc1.docx]

Supplementary Material

**Effects of vitamin D, omega-3 and a simple strength exercise programme in cardiovascular disease prevention: The DO-HEALTH randomized controlled trial**

Gängler Stephanie ^#^ , Sadlon Angélique ^#^, De Godoi Rezende Costa Molino Caroline, Willett Walter C , Manson JoAnn E, Vellas Bruno , Steinhagen-Thiessen Elisabeth, Von Eckardstein Arnold, Ruschitzka Frank, Rizzoli René, da Silva José AP, Kressig Reto W, Kanis John , Orav Endel J, Egli Andreas, Bischoff-Ferrari Heike A^*^

^#^ contributed equally

*Corresponding author, Email: [Heike.Bischoff@usz.ch](mailto:Heike.Bischoff@usz.ch)


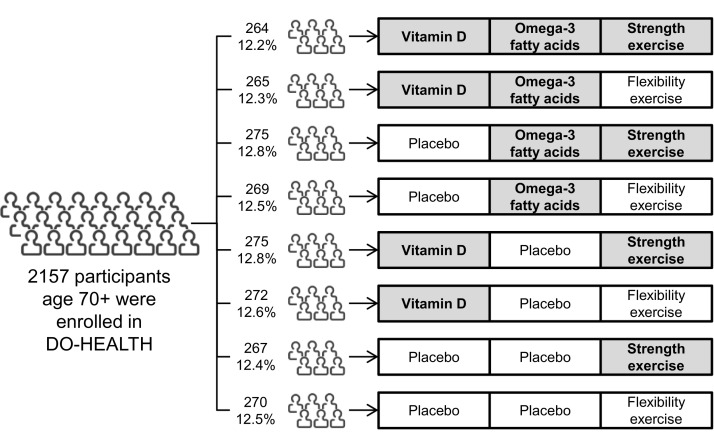


Figure S 1 Treatment allocation in DO-HEALTH. Copied from Bischoff-Ferrari HA, de Godoi Rezende Costa Molino C, Rival S, Vellas B, Rizzoli R, Kressig RW, et al. DO-HEALTH: Vitamin D3 - Omega-3 - Home exercise - Healthy aging and longevity trial - Design of a multin


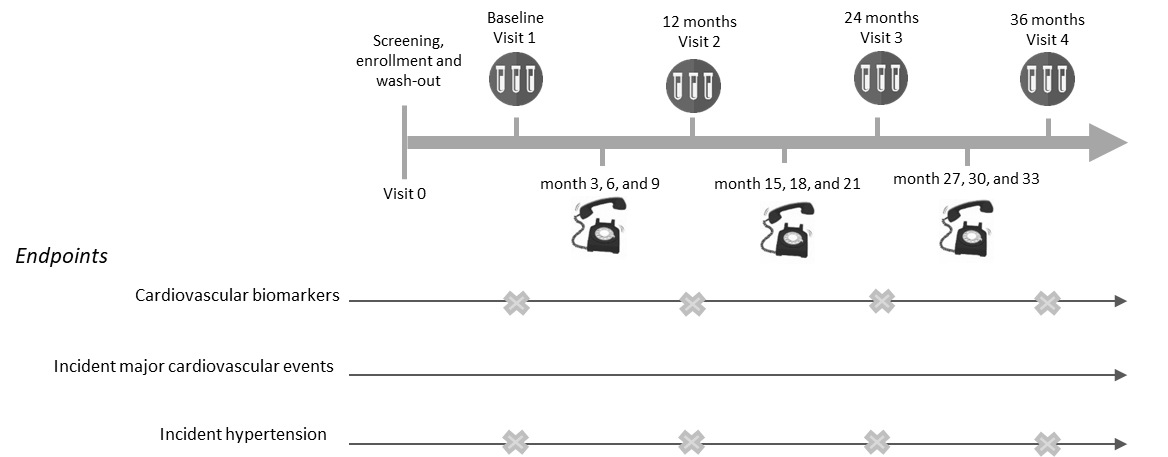


Figure S 2 Outline of the DO-HEALTH study design

Table-S 1 Population characteristics of the DO-HEALTH population used for major cardiovascular event analysis MACE analysis

|  | **Overall** | **No omega-3** | **Omega-3** | **No vitamin D_3_** | **Vitamin D_3_** | **Control Exercise** | **SHEP** |
| --- | --- | --- | --- | --- | --- | --- | --- |
|  | **N=2089** | **N=1047** | **N=1042** | **N=1049** | **N=1040** | **N=1038** | **N=1051** |
| ***Demographics*** |  |  |  |  |  |  |  |
| Age [years]^a^ | 74 [71, 77] | 74 [72, 77] § | 74 [71, 77] | 74 [71, 77] | 74 [71, 77] | 74 [71, 77] | 74 [72, 77] |
| Women, n (%) | 1302 (62.3) | 646 (61.7) | 656 (63.0) | 650 (62.0) | 652 (62.7) | 650 (62.6) | 652 (62.0) |
| Education [years] | 12.65 (4.28) | 12.68 (4.39) | 12.62 (4.18) | 12.60 (4.16) | 12.70 (4.40) | 12.64 (4.36) | 12.67 (4.20) |
| ***Clinical variables*** |  |  |  |  |  |  |  |
| BMI [kg/m^2^] | 26.29 (4.29) | 26.31 (4.33) | 26.27 (4.25) | 26.11 (4.15) | 26.47 (4.42) | 26.36 (4.37) | 26.21 (4.21) |
| Systolic BP [mmHg] | 143.51 (18.37) | 143.83 (18.52) | 143.18 (18.22) | 142.93 (18.21) | 144.09 (18.52) | 143.37 (18.30) | 143.64 (18.44) |
| Diastolic BP [mmHg] | 75.88 (10.07) | 75.97 (10.42) | 75.78 (9.71) | 75.71 (10.02) | 76.04 (10.12) | 75.82 (9.82) | 75.93 (10.32) |
| Diabetes type 2, n (%) | 144 ( 6.9) | 72 ( 6.9) | 72 ( 6.9) | 66 ( 6.3) | 78 ( 7.5) | 80 ( 7.7) | 64 ( 6.1) |
| CVD history, n (%) | 87 ( 4.2) | 47 ( 4.5) | 40 ( 3.8) | 43 ( 4.1) | 44 ( 4.2) | 39 ( 3.8) | 48 ( 4.6) |
| ***Behavioral risks*** |  |  |  |  |  |  |  |
|  |  |  |  |  |  |  |  |
| Smoking status, n (%) |  |  |  |  |  |  |  |
| Former | 645 (30.9) | 321 (30.7) | 324 (31.1) | 318 (30.3) | 327 (31.4) | 305 (29.4) | 340 (32.4) |
| Never | 1321 (63.2) | 666 (63.6) | 655 (62.9) | 669 (63.8) | 652 (62.7) | 667 (64.3) | 654 (62.2) |
| Smoker | 123 ( 5.9) | 60 ( 5.7) | 63 ( 6.0) | 62 ( 5.9) | 61 ( 5.9) | 66 ( 6.4) | 57 ( 5.4) |
| *Physical activity,* n(%) |  |  |  |  |  |  |  |
| none | 358 (17.2) | 175 (16.7) | 183 (17.6) | 157 (15.0) | 201 (19.3)# | 186 (17.9) | 172 (16.4) |
| 1-2 time per week | 628 (30.1) | 329 (31.4) | 299 (28.7) | 322 (30.7) | 306 (29.5) # | 317 (30.6) | 311 (29.6) |
| ≥3 times per week | 1101 (52.8) | 543 (51.9) | 558 (53.7) | 569 (54.3) | 532 (51.2) # | 534 (51.5) | 567 (54.0) |
| NHS_METs[METs hours/week] | 37.21 (33.25) | 36.07 (32.18) | 38.35 (34.26) | 38.99 (34.24)¶ | 35.40 (32.13) | 37.26 (32.95) | 37.15 (33.55) |
| Fat intake [g/day] | 95.50 [71.71, 124.46] | 95.32 [71.02, 123.22] | 95.64 [72.65, 125.07] | 94.54 [70.98, 125.74] | 96.61 [72.47, 123.29] | 95.17 [72.03, 124.18] | 95.76 [71.68, 124.49] |
| ***Drug intake*** |  |  |  |  |  |  |  |
| Lipid lowering drugs, n (%) |  |  |  |  |  |  |  |
| Any | 531 (25.4) | 282 (26.9) | 249 (23.9) | 247 (23.5) | 284 (27.3) | 259 (25.0) | 272 (25.9) |
| Statin | 505 (24.2) | 271 (25.9) | 234 (22.5) | 237 (22.6) | 268 (25.8) | 248 (23.9) | 257 (24.5) |
| Fibrates | 26 ( 1.2) | 10 ( 1.0) | 16 ( 1.5) | 13 ( 1.2) | 13 ( 1.2) | 12 ( 1.2) | 14 ( 1.3) |
| Bile acid sequestrant | 1 ( 0.0) | 1 ( 0.1) | 0 ( 0.0) | 1 ( 0.1) | 0 ( 0.0) | 1 ( 0.1) | 0 ( 0.0) |
| Other | 28 ( 1.3) | 12 ( 1.1) | 16 ( 1.5) | 11 ( 1.0) | 17 ( 1.6) | 14 ( 1.3) | 14 ( 1.3) |
| Anti-hypertensive drug, n(%) | 1019 (48.8) | 533 (50.9) | 486 (46.6) | 498 (47.5) | 521 (50.1) | 528 (50.9) | 491 (46.7) |
| Sex hormone use, n(%) | 136 ( 6.5) | 67 ( 6.4) | 69 ( 6.6) | 72 ( 6.9) | 64 ( 6.2) | 75 ( 7.2) | 61 ( 5.8) |
| ***Laboratory values at baseline*** |  |  |  |  |  |  |  |
| 25(OH)D<20 ng/L | 837 (40.4) | 430 (41.3) | 407 (39.4) | 425 (40.8) | 412 (40.0) | 430 (41.7) | 407 (39.1) |
| Triglycerides [mmol/]^a^ | 1.03 [0.82, 1.34] | 1.04 [0.83, 1.34] | 1.03 [0.81, 1.35] | 1.02 [0.81, 1.32] | 1.04 [0.82, 1.37] | 1.04 [0.83, 1.37] | 1.02 [0.81, 1.31] |
| Total cholesterol [mmol/L] | 5.59 (1.06) | 5.57 (1.08) | 5.62 (1.04) | 5.64 (1.06) | 5.55 (1.07) | 5.58 (1.07) | 5.61 (1.06) |
| HDL-cholesterol [mmol/L] | 1.72 (0.46) | 1.72 (0.47) | 1.72 (0.46) | 1.73 (0.46) | 1.71 (0.47) | 1.70 (0.46) | 1.74 (0.47) |
| LDL-cholesterol [mmol/L] | 3.35 (0.95) | 3.33 (0.97) | 3.38 (0.93) | 3.39 (0.94) | 3.32 (0.96) | 3.35 (0.94) | 3.35 (0.96) |
| Non-HDL cholesterol [mmol/L] | 3.88 (1.02) | 3.86 (1.04) | 3.90 (1.00) | 3.91 (1.02) | 3.85 (1.02) | 3.89 (1.02) | 3.87 (1.03) |
| Homocysteine [µmol/L]^a^ | 13.70 [11.40, 16.20] | 13.60 [11.40, 16.10] | 13.70 [11.50, 16.30] | 13.70 [11.40, 16.40] | 13.65 [11.43, 16.00] | 13.60 [11.30, 16.50] | 13.70 [11.50, 16.00] |
| NT-proBNP [ng/L]^a^ | 112.00 [66.00, 186.00] | 114.00 [66.00, 184.00] | 110.00 [67.00, 188.75] | 112.00 [67.00, 183.00] | 111.50 [66.00, 191.00] | 111.00 [66.00, 182.50] | 114.00 [66.00, 189.50] |
| Troponin T [ng/L]^a^ | 6.00 [3.97, 10.00] | 7.00 [4.03, 10.00] ∫ | 6.00 [3.92, 10.00] | 6.00 [3.97, 10.00] | 7.00 [3.97, 10.00] | 6.00 [4.00, 10.00] | 6.00 [3.94, 10.00] |
| hs-CRP[mg/L]^a^ | 1.50 [0.80, 2.90] | 1.50 [0.80, 2.90] | 1.60 [0.80, 2.98] | 1.50 [0.80, 2.92] | 1.60 [0.80, 2.80] | 1.50 [0.80, 3.00] | 1.50 [0.80, 2.80] |
| DHA [mg/L] | 77.69 (36.55) | 76.85 (36.23) | 78.54 (36.87) | 77.64 (35.83) | 77.74 (37.28) | 77.77 (37.45) | 77.61 (35.66) |
| EPA [mg/L] | 0.76 (20.52) | 30.90 (21.28) | 30.63 (19.73) | 31.03 (19.75) | 30.50 (21.27) | 31.07 (20.76) | 30.46 (20.28) |
| *Note:* Abbreviations: SHEP-Simple home based strength exercise program  a: non-normal median and interquartile range are presented and -test used to test difference between groups  b: values above 4000kcal/day for women and 5000kcal/day for men omitted as over reporting (n=51)  ¶Difference between two groups: p-value = 0.014, # Difference between two groups p-value = 0.030, § Difference between two groups p-value = 0.043, ∫Difference between two groups p-value= 0.012 •Difference between two groups p-value=0.031 | | | | | | | |


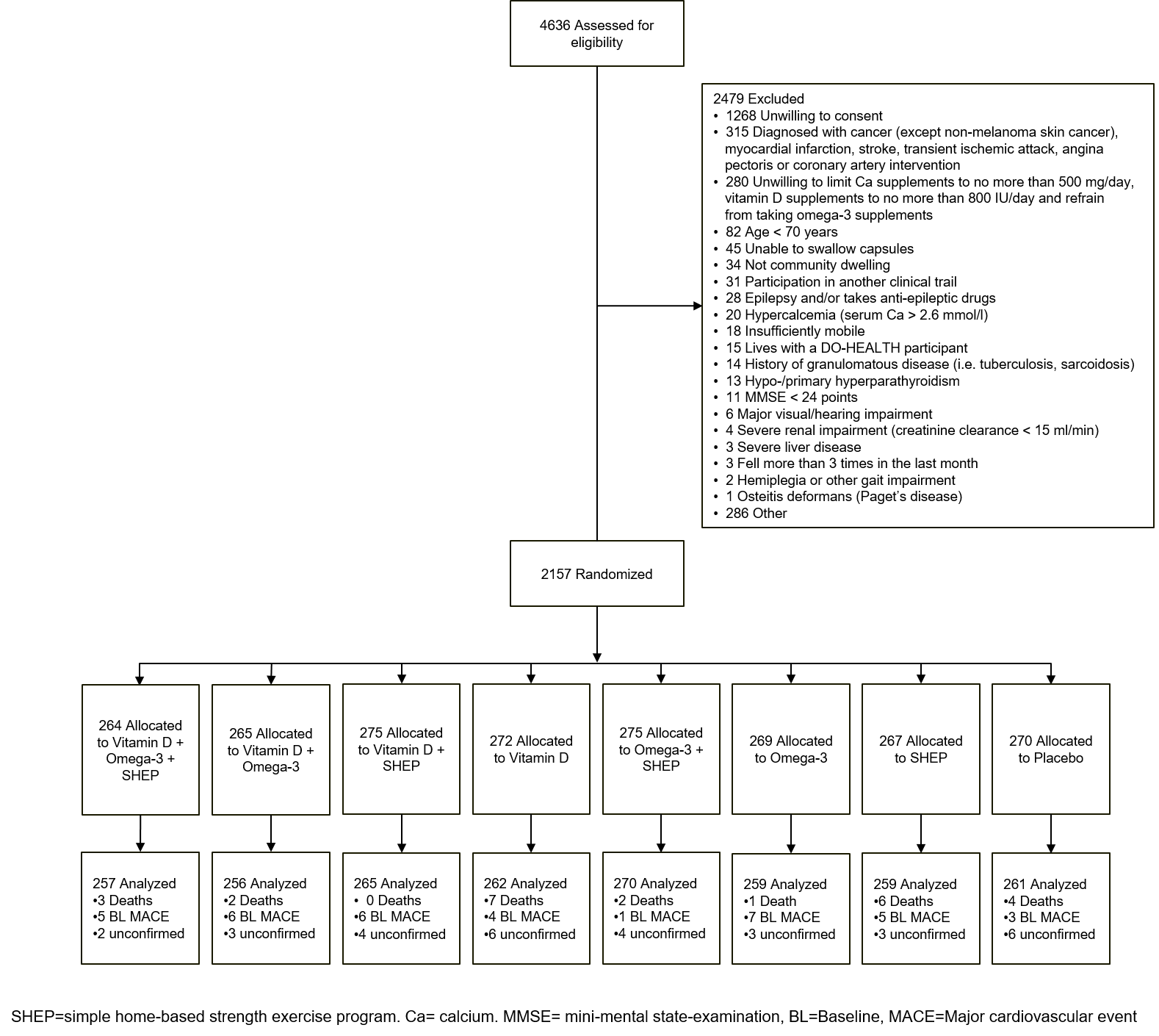


Figure-S3: Consort diagram of the major cardiovascular events (MACE) analysis

Table-S 2 Description of the home-based physical exercise program and the control exercise

| **SHEP (Strength exercise)** | **Control exercise (Flexibility exercise)** |
| --- | --- |
| 1. Sit-to-stand (quadriceps /hip extension strength training)  2. One-leg stance (hip muscles strength training plus static balance training)  3. Pull Backs against elastic resistance (seated position)  4. External shoulder rotation against elastic resistance (seated position)  5. Steps | 1. Hip and knee mobility (seated position)  2. Hip mobility (standing position)  3. Trunk and chest mobility (seated position)  4. Shoulder mobility (seated position)  5. Ankle mobility (standing position) |

Table-S 3 Adjusted least square mean change at each year by treatment group

| **Biomarkers** | **Event** | **Vitamin D_3_ (n=1069)** | **No Vitamin D_3_ (n=1072)** | **Difference (95%CI), p-value** | **Omega-3 (n=1064)** | **No Omega-3 (n=1077)** | **Difference (95%CI), p-value** | **SHEP (n=1072)** | **No SHEP (n=1069)** | **Difference (95%CI), p-value** |
| --- | --- | --- | --- | --- | --- | --- | --- | --- | --- | --- |
| **CVD risk factors** |  |  |  |  |  |  |  |  |  |  |
| Total Cholesterol [mmol/L] | Δ Year I (95%CI) | -0.04 (-0.08; 0) | -0.09 (-0.13; -0.05) | 0.05 (0; 0.11), p=0.068 | 0.01 (-0.03; 0.06) | -0.15 (-0.19;-0.11) | 0.16 (0.11; 0.22), p=0 | -0.06 (-0.1; -0.02) | -0.07 (-0.11; -0.03) | 0.01 (-0.05; 0.07), p=0.743 |
|  | Δ Year II (95%CI) | -0.08 (-0.13; -0.04) | -0.1 (-0.15; -0.06) | 0.02 (-0.04; 0.08), p=0.501 | -0.03 (-0.07; 0.02) | -0.16 (-0.21;-0.12) | 0.14 (0.08; 0.2), p=0 | -0.11 (-0.15; -0.07) | -0.08 (-0.12; -0.04) | -0.03 (-0.09; 0.03), p=0.369 |
|  | Δ Year III (95%CI) | -0.15 (-0.2; -0.11) | -0.19 (-0.23; -0.15) | 0.04 (-0.02; 0.1), p=0.246 | -0.1 (-0.14; -0.05) | -0.25 (-0.29;-0.21) | 0.15 (0.09; 0.21), p=0 | -0.18 (-0.22; -0.13) | -0.17 (-0.21; -0.13) | -0.01 (-0.07; 0.05), p=0.821 |
| HDL-cholesterol [mmol/L] | Δ Year I (95%CI) | 0.05 (0.03; 0.06) | 0.05 (0.04; 0.07) | -0.01 (-0.03; 0.01), p=0.372 | 0.09 (0.08; 0.11) | 0.01 (-0.01;0.02) | 0.09 (0.06; 0.11), p=0 | 0.05 (0.04; 0.07) | 0.05 (0.03; 0.06) | 0 (-0.02; 0.03), p=0.635 |
|  | Δ Year II (95%CI) | 0.06 (0.04; 0.07) | 0.07 (0.05; 0.08) | -0.01 (-0.03; 0.01), p=0.328 | 0.1 (0.09; 0.12) | 0.02 (0.01;0.04) | 0.08 (0.06; 0.1), p=0 | 0.06 (0.05; 0.08) | 0.06 (0.05; 0.08) | 0 (-0.02; 0.02), p=0.973 |
|  | Δ Year III (95%CI) | 0.02 (0; 0.03) | 0.02 (0.01; 0.04) | -0.01 (-0.03; 0.02), p=0.519 | 0.06 (0.04; 0.07) | -0.02 (-0.03;0) | 0.08 (0.05; 0.1), p=0 | 0.02 (0.01; 0.04) | 0.02 (0; 0.03) | 0.01 (-0.01; 0.03), p=0.476 |
| LDL-cholesterol [mmol/L] | Δ Year I (95%CI) | -0.08 (-0.11; -0.04) | -0.13 (-0.17; -0.09) | 0.05 (0; 0.1), p=0.047 | -0.05 (-0.08; -0.01) | -0.16 (-0.2;-0.13) | 0.12 (0.07; 0.17), p=0 | -0.1 (-0.14; -0.07) | -0.11 (-0.14; -0.07) | 0 (-0.05; 0.05), p=0.901 |
|  | Δ Year II (95%CI) | -0.14 (-0.18; -0.1) | -0.16 (-0.2; -0.12) | 0.02 (-0.03; 0.08), p=0.395 | -0.1 (-0.14; -0.06) | -0.2 (-0.24;-0.16) | 0.1 (0.05; 0.15), p=0 | -0.17 (-0.21; -0.13) | -0.14 (-0.17; -0.1) | -0.03 (-0.08; 0.02), p=0.242 |
|  | Δ Year III (95%CI) | -0.19 (-0.23; -0.15) | -0.23 (-0.27; -0.19) | 0.04 (-0.01; 0.09), p=0.145 | -0.15 (-0.19; -0.12) | -0.27 (-0.31;-0.23) | 0.12 (0.06; 0.17), p=0 | -0.22 (-0.26; -0.18) | -0.2 (-0.24; -0.16) | -0.02 (-0.07; 0.03), p=0.466 |
| Non-HDL cholesterol [mmol/L] | Δ Year I (95%CI) | -0.08 (-0.12; -0.04) | -0.15 (-0.19; -0.11) | 0.06 (0.01; 0.12), p=0.023 | -0.08 (-0.12; -0.04) | -0.16 (-0.19;-0.12) | 0.08 (0.03; 0.14), p=0.004 | -0.11 (-0.15; -0.07) | -0.12 (-0.16; -0.08) | 0 (-0.05; 0.06), p=0.921 |
|  | Δ Year II (95%CI) | -0.14 (-0.18; -0.1) | -0.18 (-0.22; -0.14) | 0.04 (-0.02; 0.09), p=0.227 | -0.13 (-0.17; -0.09) | -0.19 (-0.23;-0.15) | 0.06 (0; 0.12), p=0.034 | -0.17 (-0.21; -0.13) | -0.14 (-0.19; -0.1) | -0.03 (-0.09; 0.03), p=0.328 |
|  | Δ Year III (95%CI) | -0.17 (-0.21; -0.13) | -0.22 (-0.26; -0.17) | 0.04 (-0.01; 0.1), p=0.138 | -0.15 (-0.2; -0.11) | -0.23 (-0.27;-0.19) | 0.08 (0.02; 0.14), p=0.009 | -0.2 (-0.24; -0.16) | -0.19 (-0.23; -0.14) | -0.02 (-0.07; 0.04), p=0.605 |
|  |  | Vitamin D (n=272) | Placebo (n=269) | Difference (95%CI) | Omega-3(n=266) | Placebo (n=269) | Difference (95%CI) | SHEP (n=264) | Placebo (n=269) | Difference (95%CI) |
| Triglycerides [mmol/L]^a^ | Δ Year I (95%CI) | 0.02 (-0.03; 0.06) | -0.02 (-0.06; 0.03) | 0.03 (-0.03; 0.1), p=0.294 | -0.08 (-0.13; -0.04) | -0.02 (-0.06; 0.03) | -0.07 (-0.13; 0), p=0.042 | 0 (-0.05; 0.04) | -0.02 (-0.06; 0.03) | 0.01 (-0.05; 0.08), p=0.664 |
|  | Δ Year II (95%CI) | 0.01 (-0.03; 0.06) | 0.04 (-0.01; 0.08) | -0.02 (-0.09; 0.04), p=0.48 | -0.05 (-0.1; -0.01) | 0.04 (-0.01; 0.08) | -0.09 (-0.15; -0.02), p=0.007 | 0.02 (-0.03; 0.06) | 0.04 (-0.01; 0.08) | -0.02 (-0.08; 0.04), p=0.534 |
|  | Δ Year III (95%CI) | 0.08 (0.03; 0.12) | 0.09 (0.05; 0.14) | -0.02 (-0.08; 0.05), p=0.621 | 0.01 (-0.04; 0.05) | 0.09 (0.05; 0.14) | -0.08 (-0.15; -0.02), p=0.011 | 0.09 (0.04; 0.14) | 0.09 (0.05; 0.14) | 0 (-0.07; 0.06), p=0.991 |
| **CVD risk markers** |  |  |  |  |  |  |  |  |  |  |
| hs-CRP [mg/L] | Δ Year I (95%CI) | -0.18 (-0.48; 0.13) | -0.11 (-0.42; 0.19) | -0.06 (-0.49; 0.36), p=0.772 | -0.2 (-0.51; 0.11) | -0.09 (-0.4;0.21) | -0.1 (-0.53; 0.32), p=0.636 | -0.1 (-0.4; 0.21) | -0.19 (-0.5; 0.11) | 0.1 (-0.33; 0.52), p=0.654 |
|  | Δ Year II (95%CI) | 0.27 (-0.14; 0.69) | -0.05 (-0.47; 0.36) | 0.32 (-0.26; 0.91), p=0.276 | 0.08 (-0.34; 0.5) | 0.14 (-0.27;0.56) | -0.07 (-0.65; 0.52), p=0.826 | 0.22 (-0.2; 0.64) | 0 (-0.42; 0.42) | 0.22 (-0.37; 0.8), p=0.463 |
|  | Δ Year III (95%CI) | -0.07 (-0.4; 0.26) | -0.32 (-0.65; 0.01) | 0.25 (-0.21; 0.71), p=0.284 | -0.16 (-0.5; 0.17) | -0.23 (-0.56;0.1) | 0.07 (-0.39; 0.53), p=0.773 | -0.21 (-0.54; 0.12) | -0.19 (-0.52; 0.14) | -0.02 (-0.48; 0.45), p=0.947 |
| NT-ProBNP [ng/L] | Δ Year I (95%CI) | 19.43 (4.56; 34.3) | 16.39 (1.63; 31.15) | 3.04 (-17.34; 23.42), p=0.77 | 17.8 (2.77; 32.82) | 18.02 (3.41;32.63) | -0.22 (-20.63; 20.18), p=0.983 | 23.99 (9.11; 38.87) | 11.83 (-2.92; 26.59) | 12.15 (-8.24; 32.55), p=0.243 |
|  | Δ Year II (95%CI) | 55.39 (34.12; 76.67) | 33.17 (11.92; 54.42) | 22.22 (-7.46; 51.9), p=0.142 | 48.97 (27.44; 70.5) | 39.59 (18.58;60.6) | 9.38 (-20.32; 39.08), p=0.536 | 49.3 (28.02; 70.58) | 39.27 (18.01; 60.52) | 10.03 (-19.66; 39.72), p=0.508 |
|  | Δ Year III (95%CI) | 78.95 (56.38; 101.53) | 50.88 (28.33; 73.43) | 28.07 (-3.46; 59.61), p=0.081 | 60.42 (37.61; 83.23) | 69.41 (47.09;91.74) | -8.99 (-40.54; 22.55), p=0.576 | 72.95 (50.36; 95.54) | 56.88 (34.35; 79.42) | 16.07 (-15.46; 47.6), p=0.318 |
| Troponin [ng/L] | Δ Year I (95%CI) | 2.02 (1.35; 2.69) | 1.88 (1.22; 2.54) | 0.14 (-0.78; 1.07), p=0.76 | 2.07 (1.4; 2.75) | 1.83 (1.17;2.49) | 0.24 (-0.68; 1.17), p=0.606 | 1.67 (1; 2.33) | 2.24 (1.57; 2.9) | -0.57 (-1.49; 0.35), p=0.226 |
|  | Δ Year II (95%CI) | 2.53 (1.58; 3.49) | 2.55 (1.6; 3.5) | -0.01 (-1.35; 1.32), p=0.983 | 2.56 (1.59; 3.52) | 2.53 (1.59;3.47) | 0.03 (-1.31; 1.36), p=0.966 | 2.5 (1.55; 3.45) | 2.59 (1.63; 3.54) | -0.09 (-1.42; 1.25), p=0.898 |
|  | Δ Year III (95%CI) | 4.14 (2.57; 5.71) | 3.69 (2.12; 5.25) | 0.46 (-1.75; 2.66), p=0.686 | 3.3 (1.72; 4.88) | 4.53 (2.97;6.08) | -1.23 (-3.44; 0.98), p=0.276 | 4.19 (2.62; 5.75) | 3.64 (2.07; 5.21) | 0.55 (-1.66; 2.76), p=0.625 |
| Note: Models were adjusted for baseline biomarker concentration, visit, baseline BMI and change of BMI from baseline, prior fall, sex, study site, age, and a spline at age 85. The change yearly change was calculated using the interaction between treatment and visit. The overall change across three years was assessed without interaction, due to lack of significance of the interaction term. ^a^For Triglycerides there was a significant treatment interaction (p<0.1). Therefore, we included treatment indicators for each of the 8 combinations of treatments in the regression models and each intervention group (with neither of the other two interventions present) was compared to the 270 participants who received none of the 3 intervention. | | | | | | | | | | |


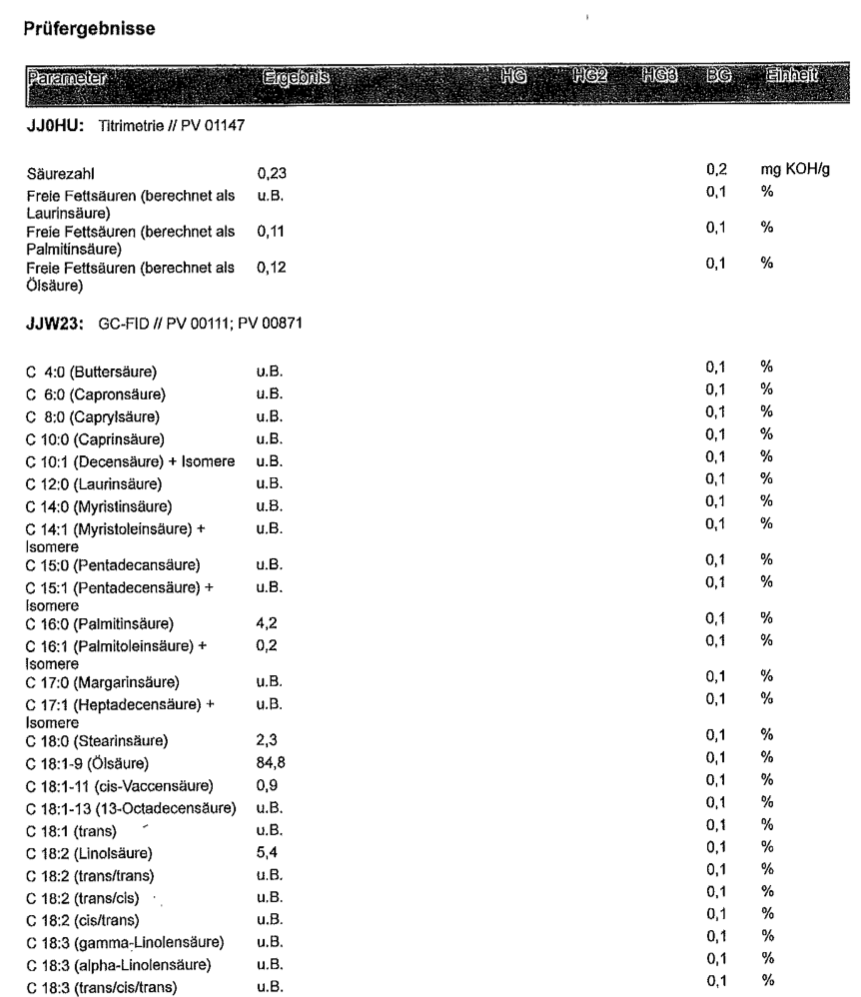


Figure S 4 Composition of the placebo used in DO-HEALTH

Table-S 4 Methodological parameters of biomarker measurements

| Parameter | Method | Analytical Device | Limit of Detection | Unit | Calibrated Range | Unit | Coefficient of variation |
| --- | --- | --- | --- | --- | --- | --- | --- |
| Total cholestrol | Enzymatic color test (CHOD-POD Test) | Roche Cobas c701 | 0.1 | mmol/L | 0.1 - 20.7 | mmol/L | 1.8% at at2.8mmol/L |
|  |  |  |  |  |  |  | 1.4% at 4.6mmol/L |
| Triglyceride | Enzymatic color test | Roche Cobas c701 | 0.1 | mmol/L | 0.1 - 10.0 | mmol/L | 1.1% at 1.01mmol/L |
|  |  |  |  |  |  |  | 0.9% at 1.91mmol/L |
| HDL | Enzymatic color test | Roche Cobas c701 | 0.08 | mmol/L | 0.08 - 3.12 | mmol/L | 1.2% at 0.76mmol/L |
|  |  |  |  |  |  |  | 1.2% at 1.59mmol/L |
| LDL | Calculation from total cholesterol, HDL, Triglyceride |  |  |  |  |  |  |
| NT-proBNP | Elektro-Chemilumineszenz-Immunoassay | Roche Cobas e602 | 5 | ng/L | 5 - 35000 | ng/L | 4.4% at 126.8ng/L |
|  |  |  |  |  |  |  | 3.0% at 4449.6ng/L |
| hs-CRP | particle enhanced turbidimetric immunoassay | Roche Cobas c701 | 0.3 | mg/L | 0.3 - 350 | mg/L | 2.4% at 6.5mg/L |
|  |  |  |  |  |  |  | 2.8% at 32.5mg/L |
| Troponin T | ECLIA | Roche Cobas e602 | 0.005 | µg/L | 0.003 - 10 | µg/L | 3.2% at 0.025µg/L |
|  |  |  |  |  |  |  | 1.8% at 2.052µg/L |
